# Anorexic and metabolic effect of jojoba: potential treatment against metabolic syndrome and hepatic complications

**DOI:** 10.1186/s12986-020-00441-3

**Published:** 2020-03-30

**Authors:** Sahla Belhadj, Stephanie Dal, Fakhreddine Khaskhoussi, Elisa Maillard-Pedracini, Olfa Hentati, Séverine Sigrist

**Affiliations:** 1 UMR DIATHEC, EA 7294, Federation of Traditional Medicine of Strasbourg (FMTS), University of Strasbourg, Strasbourg, France; 2 AGRO-CRC, Al Amine Al Abbassi Street, 1002 Tunis, Tunisia; 3 Institut Supérieur de Biotechnologie de Sfax, Université de Sfax, Route de Soukra, Km 4, BP 1175, 3038 Sfax, Tunisia; 4 Laboratoire Génie Environnement et Ecotechnologie, Ecole Nationale d’Ingénieurs de Sfax (LGEET LR16ES19-ENIS), Université de Sfax, Route de Soukra, Km 4, BP 1173, 3038 Sfax, Tunisia

**Keywords:** Hight fat Hight fat diet, Metabolic syndrome, Oxidative stress, Jojoba

## Abstract

**Background:**

Evaluation of the action of various traditional plants to treat metabolic syndrome are strongly studied. In our study, we investigated the effect of the Tunisian jojoba seed on a metabolic syndrome induced in rat by the High Fat diet and High Fructose (HFHF) and its renal and hepatic complications.

**Methods:**

The rats were fed with HFHF or Normal Diet (ND) for a period of 8 weeks. After that, a switch from HFHF to ND or Normal Diet Jojoba (NDJ),(jojoba diet approach) or High Fat and High Fructose and Jojoba diet (HFHFJ) (nutraceutical approach) has been done. Metabolic disorder was evaluated by measuring the fasting body weight, glycemia and C-peptide and leptin. Oxidative stress parameters like ThioBarbituric Acid Reactive Substances (TBARS) and Total Antioxidant Capacity (TAOC) were analyzed in the plasma and renal and hepatic function were determined by the measure of creatinine and alanine transferase (ALT) respectively. Histological analysis was performed on the liver, kidney and pancreas.

**Results:**

HFHF diet exhibited characteristics of metabolic syndrome presented by insulin resistance, hyperinsulinemia, hyperleptinemia, fat mass with hepatic steatosis and renal disorder. HFHF diet was associated with oxidative stress (OS) presented by an increase in TBARS and a decrease in TAOC. Adding jojoba seeds to HFHF rat group diet induced a decrease in body weight, fat mass (58 and 41%), insulin resistance (59 and 56%), oxidative stress (60 and 41%), liver steatosis (from a score = 3 to a score = 0) and renal complications (25 and 42%). This effect was emphasized with diet approach.

**Conclusion:**

The results demonstrated the beneficial effect of jojoba against metabolic syndrome and oxidative stress, suggesting that jojoba could be used in the prevention and treatment of metabolic syndrome.

**Graphical abstract:**

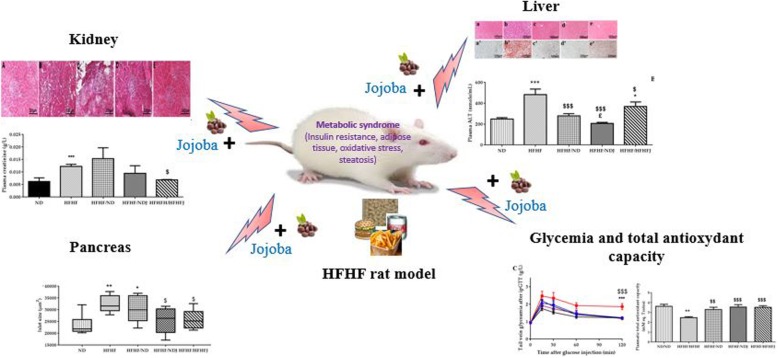

## Background

The current context was dominated by over-consumption of food and a sedentary lifestyle. These allow to set up the metabolic syndrome causing terrible consequences on human health [1, 2]. Accumulating evidence indicates that obesity is closely associated with an increased risk of metabolic diseases such as insulin resistance (IR), type 2 diabetes (DT2), dyslipidemia and nonalcoholic fatty liver disease. This metabolic syndrome is defined according to the International Diabetes Federation as a set of metabolic abnormalities such as insulin resistance, nonalcoholic fatty liver disease (NAFLD), glucose intolerance, obesity and type 2 diabetes [3]. The hypertrophy of adipose tissue leads to an increase in leptin secretion which in turn increases adipocyte lipolysis and IR [4]. Leptin is almost exclusively expressed and produced by adipose tissue.

Several studies have also documented a strong relationship between hepatic steatosis and IR [5, 6]. In the first stage, the metabolic syndrome causes a benign steatosis of the liver. At a later stage, the metabolic syndrome is responsible for a steatosis associated with inflammation, which is called non-alcoholic steatohepatitis (NASH) [7].

Moreover, there are clinical evidences of a strong association between metabolic syndrome and chronic diseases. Chen et al. [8], in analyzing The Third National Health and Nutrition Examination Survey database results on more than 6000 adults, found that the risk of microalbuminuria and chronic kidney disease was significantly higher in patients with metabolic syndrome and that this risk increased progressively with the number of components of the syndrome [9].

Metabolic syndrome is often associated to OS. In fact, there is an unbalance between the reactive oxygen species (ROS) and the antioxidant defense system in favor of these ROS [10]. Oxidative stress plays a critical role in the installation of this metabolic syndrome, which is manifested by several diseases such as hypertension and DT2 [11]. It is also linked to insulin and adiposity resistance [12] suggesting that OS may be an early event in the development of these pathologies and not the consequence of these chronic diseases [10].

Numerous studies have confirmed the strong association between diet rich in plant foods and health [13]. The positive effects of these foods may rely on their content on phytochemicals, antioxidant vitamins and fibers. Most of these dietary compounds contribute to a well redox balance by several mechanisms, such direct scavenging or neutralization of free radicals, modulation of enzyme activity and expression, and anti-inflammatory action [14, 15].

*Simmondsia californica* (also called *Simmondsia chinensis* or jojoba), a shrub of the family Buxaceae (or Simmondsiaceae) was found in the desert regions of the southeastern United States. The oil obtained from the Jojoba seeds has many applications [16]Once the oil has been extracted, there remains a farinaceous residue (meal) containing between 25 and 30% of protein [16]. Incorporation of this residue into rat feed causes a significant decrease in weight due to loss of appetite. This is related to the presence of Simmondsin in meals. An addition of this product to the diet of the animal resulted in the discontinuation of feeding, whereas its oral toxicity is low (LD50 > 4 g /kg) [17].

In a recent study [18], we have demonstrated that extraction of jojoba seeds by distilled water endorses different classes of polyphenols such as hydroxybenzoic acid, flavonoids, anthocyanins and hydroxycinnamic acids. For the first time, this study showed hypoglycemic and antioxidant effects, of simmondsin and aqueous extract, on Rin m5f cells.

The aim of this study was to evaluate the effect of Tunisian jojoba seeds on HFHF diet induced metabolic syndrome in Wistar rats and its consequence in liver and kidney complications.

## Methods

### Animals’ preparation

This study has been done in compliance with the “Guide to the Care and Use of Laboratory Animals” published by the National Institute of Health of the United States (NIH publication No. 85–23, revised 1996), and the current protocol has been adopted and approved by the local Ethics Committee (Comité Régional d’Ethique en Matière d’Expérimentation Animale CREMEAS, Strasbourg, France, CEEA-35). All efforts have been made to minimize the suffering of animals and to reduce the number of used animals**.**

Thirty male Wistar rats (Janvier Labs, Le Genest-Saint-Isle, France) weighing 185–200 g upon arrival were housed in a climate-controlled room (22 ± 2 °C and 60% relative humidity) and kept in a 12-h light/dark cycle with food and water ad libitum.

A rat model made intolerant to glucose by a dietary approach was developed in the laboratory [19, 20]. Twenty-four rats were fed with High Fat Diet/High Fructose (HF/HF). High Fat diet composition was 21.4% fat; 17.5% protein; 50% carbohydrates; 3.5% fiber (Special Diets Services, Saint Gratien, France) and high fructose was added in drinking water (25% fructose (Sigma-Aldrich, Saint-Louis, United States). Six control groups were fed with ND consisting of 3.1% fat, 16.1% protein, 3.9% fiber, and 5.1% ash and 57.5% carbohydrate (2.9% Kcal / g) (ND) (SAFE, Augy, France) with water ad libitum.

After 2 months of High fat diet HFHF to induce metabolic syndrome, HFHF rats were randomly divided into four groups (*n* = 6/group) for two more months: HFHF or HFHF with Jojoba supplementation at 3% (J) (HFHF/HFHFJ) represented “nutraceutical approach”; changed to normal diet, plus water (HFHF/ND), or ND plus J (3%) (HFHF/NDJ) represented “lifestyle measures,” in comparison to ND rats. The HFHF groups was then divided in several groups (*n* = 6/group) with different: a group with the maintenance of the HFHF diet, a group of 6 rats or the HFHF diet is supplemented with jojoba (HFHF-HFHFJ) nutraceutical approach, and finally the HFHF diet is replaced by a normal diet with (HFHF -ND) or without Jojoba (HFHF-NDJ) (an explanatory diagram is given in supplementary data S1).

Five kilograms of jojoba seeds were previously collected from Meknessi fields (Sidi Bouzid, Tunisia) in August 2015. Jojoba seeds were then crushed and incorporated in both diets (ND and HFD) at 3% for oral intake (SAFE for ND and SDS for HFD).

Throughout the experimental period, the body weights of the rats as well as the food intake were checked weekly. All the rats were sacrificed at the end of the study.

### Sacrifice

Before anesthesia, each rat was weighed, its capillary blood glucose was measured with a glucose meter (AccuChek®, Roche, Basel, Switzerland), and a blood sample was taken from the tail to measure metabolic parameters. Then, the rats were anesthetized with intraperitoneal pentobarbital sodium (Centravet, France) injection (50 mg/kg). Blood was then collected in the abdominal aorta on lithium heparinate (Greiner Bio One, Les Ulis, France) or on dry tubes. The plasma and serum samples were stored at − 80 °C after centrifugation (4 °C, 2 min, 10.000×g) to determine the biochemical and oxidative parameters. The pancreas, the liver, the kidney and the fat were harvested. The fat and the liver were weighed. A piece of each organ was placed in the Tissue-Tek® (Optimal Cutting Temperature Compound, Electron Microscopy Sciences, Hatfield, PA, USA) and frozen in liquid nitrogen and then stored at − 80 °C to determine the histological parameters.

### Plasmatic metabolic parameters

Intraperitoneal glucose tolerance test (IpGTT) was performed on fasted rats before the beginning of the study and 4 months after. Capillary glucose was measured using the glucometer at 0, 15, 30, 60, and 120 min after the intraperitoneal injection of 2 g glucose/kg (Fisher, Leicestershire, USA). Blood samples were harvested from the tail at 0 and 60 min to measure plasma glucose (g/L) by the RTU® glucose method (Biomérieux, Graponne, France). C- peptide secretion (pmol/L) was determined using Rat C-peptide ELISA (Mercodia, Uppsalla, Sweden). IR was determined by calculating the Homeostasis Model Assessment Indexes-Insulin Resistance (HOMA2-IR) using the HOMA-IR model calculator using fasting glucose and fasting C-peptide (HOMA 2, http://www.dtu.ox.ac.uk/homa) . If HOMA2 is higher than 2.4, the IR was confirmed.

The measurement of fructosamine has utility to know retrospectively (2–3 weeks) the level of glucose concentration in blood. The levels of Fructosamine (μmol/L) was determined using using the Fructosamine NBT Kinetic kit (SPINREACT, Spain) according to the manufacturer’s protocol.

Fasting leptin assay was measured by ELISA kit (Millipore, Billerica, MA, USA). Results were expressed by ng/mL.

ALT level was measured by a fluorimetric assay kit (Elisa kit, Sigma-Aldrich, USA) according to the manufacturer’s instruction and expressed in nmol/mL. The creatinine concentration was also carried out by a fluorimetric method creatinine assay kit (Elisa kit, Abcam, Paris, France) and the results were expressed in g/L.

### Plasma markers of oxidative stress

Lipid peroxidation was estimated by the TBAR assay kit (OxiSelect™ TBARS Assay Kit-MDA Quantitation, Cell Biolabs Inc., San Diego, CA, USA). This assay consists in quantifying malondialdehyde (MDA), an alkylating agent derived from the degradation of polyunsaturated lipids by ROS. Results were presented in μM/MDA. TAOC with the radical cation 2,2′-azino-bis (3-ethylbenzothiazoline-6-sulphonic acid (ABTS^•+^) was achieved by a 3,4-dihydro-6-hydroxy-2,5,7,8-tetramethyl-2H-1-benzopyran-2-carboxylic acid (Trolox) equivalent antioxidant capacity method described by Auberval et al., 2014 [19]. TAOC was presented by mM eq. Trolox.

### Histological analysis

The degree of hepatic, pancreatic and renal histological changes was assessed on 10 μm cryosections fixed with 4% paraformaldehyde by eosin/hematoxylin coloration (pancreas and kidney) and Oil Red O staining (pancreatic steatosis). The islet surface distribution was determined in the pancreas. At least three sections were examined per animal. The islet surface area was measured using Nikon NIS Elements Br software (Nikon, Tokyo, Japan), and the distributions were reported using box and whiskers plots with median, first and third quartile and extreme values.

In the liver, the degree of steatosis was defined on sections according to the Standard Kleiner classification (degree of lipid droplet in hepatocytes) [21]. The degree of steatosis was assessed as follows: 0 (less than 5%), 1 (between 5 and 33%), 2 (between 33 and 66%) and 3 (higher than 66%), complicated or not by fibrosis. No accumulation of lipids occurs in the pancreas and the kidney.

### Statistical analysis

Values are expressed as means ± standard error of mean (SEM), and n indicates the number of rats per group. Statistical analysis was performed analysis of variance (ANOVA) followed by Tukey’s least significant difference (LSD) test after normality test validation (Statistica® version 12, StatSoft, France). Six animals were analyzed in each group for metabolic studies and three animals were analyzed per group for the histological studies. Differences were considered statistically significant at *p*-value < 0.05 (*, $), *p*-value < 0.01 (**, $$), or *p*-value < 0,001 (***, $$$).

## Results

### Effect of jojoba meal on weight

**Fig. 1:**
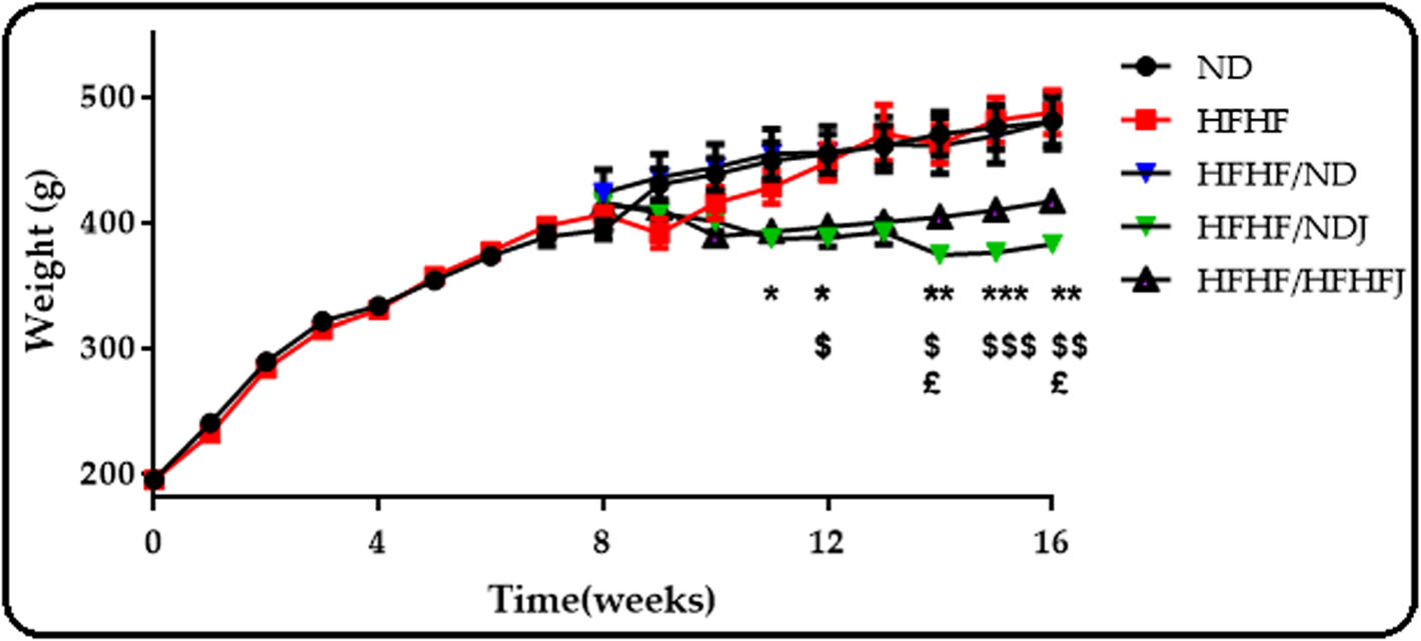
Body weight of rats after 16 weeks. The data are expressed as the mean ± SEM with several animals per group (*n* = 6)., * significant difference between HFHF/ND and HFHF/NDJ, $ significant difference between HFHF and HFHF/HFHFJ, £ significant difference between HFHF/ND and HFHF/HFHFJ. All values are indicative of mean ± SEM. *p < 0.05, ** *p* < 0.01, *** *p* < 0.001.

As shown in Fig. 1, from the beginning to the end of treatment, there was no significant difference between the groups treated with ND and HFHF diet. On the other hand, jojoba meal produced a significant decrease on body weight. Indeed, the rat weights in the two jojoba groups were statistically different for the ND and HFHF groups (*p* < 0.05).

### Effect of jojoba meal on glycaemia

**Fig. 2:**
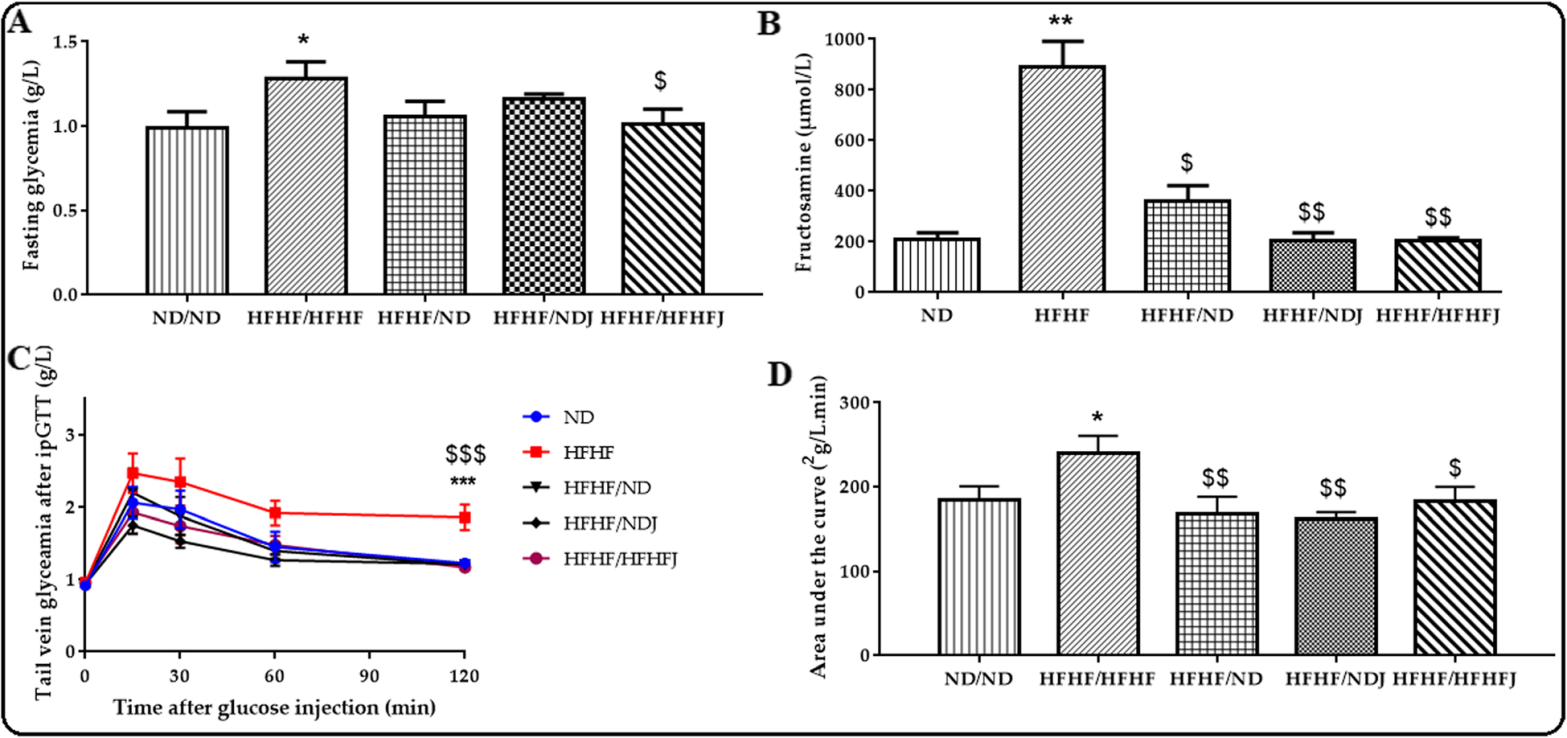
Effect of jojoba meal on glucose metabolic of rats after eight weeks of HFHF diet. Glycaemia (**a**) and fructosamine (**b**), under fasting and refeeding conditions, glucose tolerance test (IpGTT) (**c**) and (IpGTT) area under the curve (**d**). *significant difference compared to ND rats, $ significant difference compared to HFHF rats.. All values are indicative of Mean ± SD. *p < 0.05, ** *p* < 0.01, *** *p* < 0.001.

As shown in Fig. 2a, after 4 months, there was a significant difference on fasting glycemia between ND (1.02 g/L ± 0.10) and HFHF (1.28 g/L ± 0.10, *p* < 0.05) rats. The switch from HFHF diet to ND or NDJ maintained a fasting glycaemia comparable to ND alone (1.06 g/L ± 0.08 and 1.16 g/L ± 0.03 respectively) but no statistical difference was observed with HFHF. On the other hand, addition of the jojoba to HFHF diet induced a significant decreased of glycemia compared to the HFHF rats (1.00 g/L ± 0.09, *p* < 0.05). HFHF diet increased also significantly fructomanine levels (212.29 μmol/L ± 19.40 (ND), vs. 849.25 μmol/L ± 69.30 (HFHF); *p* < 0.01). On the contrary, the switch to ND reduced significantly fructosamine levels (360.89 μmol/L ± 48.60, *p* < 0.05). Moreover, the switch to NDJ and HFHFJ improved this decrease (203.20 μmol/L ± 27.70 and 202.49 μmol/L ± 7.44, *p* < 0.01), (Fig. 2b).

Finally, HFHF diet increased significantly (*p* < 0.05) glucose intolerance in rats (AUC IpGTT: 184.5 ± 16.09 (ND) vs. 239.55 ± 21.14 (HFHF)). The nutraceutical or diet approach, significantly reduced this glucose intolerance after 2 h of IpGTT (AUC IpGTT: 168.32 ± 20.03, 162.52 ± 7.69, 182.50 ± 17.69 for HFHF/ND, HFHF/NDJ and HFHF/HFFHJ rat groups, respectively), (Fig. 2c and Fig. 2d).

### Effect of jojoba meal on C-peptide and HOMA2-IR

**Fig. 3:**
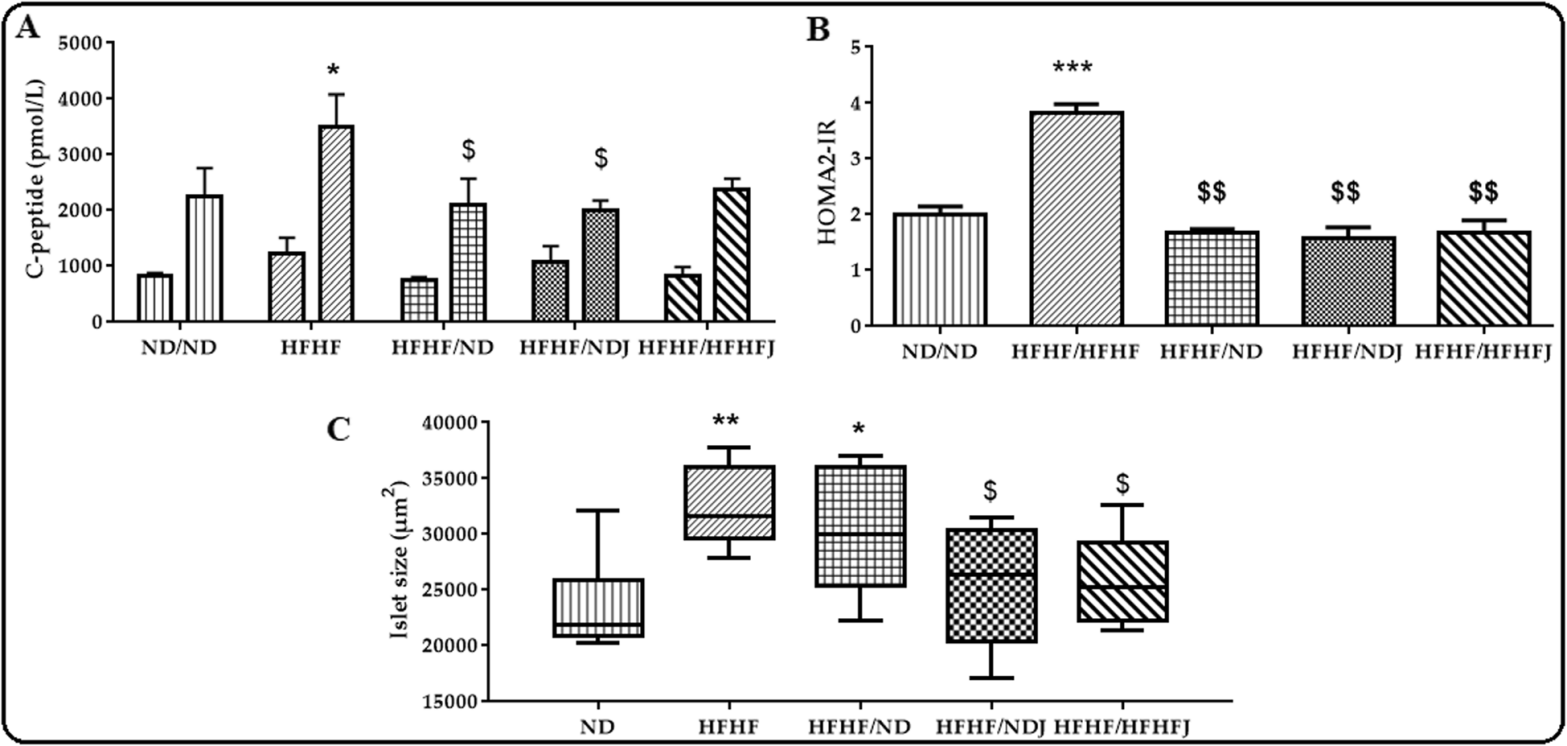
Effect of jojoba meal on C-peptide (**a**), HOMA2-IR (**b**) and islet size (**c**). *significant difference compared to ND rats, $ significant difference compared to HFHF rats.. All values are indicative of Mean ± SD. *p < 0.05, ** *p* < 0.01, *** *p* < 0.001.

As shown in Fig. 3a, there was no difference between groups for fasting C-peptide. On the contrary, HFHF diet increased significantly C-peptide after refeeding (2099 ± 433.42 (ND) vs. 3492 ± 583.01 pmol/L (HFHF); *p* < 0.05). The switch to ND and NDJ significantly (*p* < 0.05) decreased the C-peptide level. In contrast, there was no difference after the switch to HFHFJ.

Moreover, the HOMA2-IR (Fig. 3b) was significantly increased in HFHF diet (3.83 ± 0.12; *p* < 0.01) compared to ND (2.00 ± 0.12). HOMA2 of the HFHF group was higher than 2.4 confirming insulin resistance. The switch on nutraceutical or on diet approach restored normal HOMA-IR state (1.68 ± 0.04;1.58 ± 0.16 and 1.68 ± 0.18 for HFHF/ND, HFHF/NDJ and HFHF/HFHFJ rats groups, respectively).

The histology of the pancreas showed a preservation of islets structure in the different rat groups. Indeed, the islets presented an ovoid form and a cytoplasm of pink purple color with nuclei colored in black. However, islets size was significantly increased in HFHF group (*p* < 0.01) even after 2 months of ND alone (HFHF/ND, *p* < 0.05). Addition of Jojoba (HFHF/HFHFJ, HFHF/NDJ) significantly decrease islet size (p < 0,05) compared to HFHF.

### Effect of jojoba meal on leptin and fat weight

**Fig. 4:**
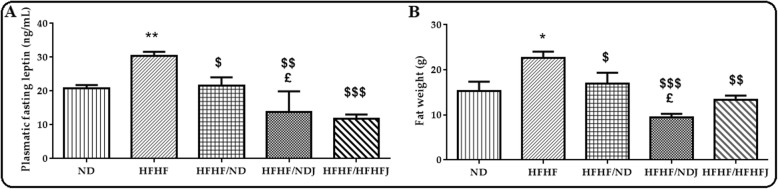
Effect of jojoba meal on leptin and fat weight. *significant difference compared to ND rats, $ significant difference compared to HFHF rats.. ^£^significant difference compared between HFHF/ND rats and HFHF/NDJ rats.. All values are indicative of Mean ± SD. *p < 0.05, ** *p* < 0.01, *** *p* < 0.001.

HFHF diet induced a significant increase on plasmatic leptin (20.78 ng/mL ± 0.66 vs. 30.40 ng/mL ± 0.66, *p* < 0.001)). Conversely, the levels of leptin significantly decreased with ND, NDJ and HFHFJ (Fig. 4a). The rise in leptin was associated with a significant expansion of fat in HFHF rats (*p* < 0.05) while the decrease of leptin in the other rat groups (HFHF/ND, HFHF/NDJ and HFHF/HFHFJ) was correlated by a significant decrease in fat storage (Fig. 4b).

### Effect of jojoba meal on liver complications

**Fig. 5:**
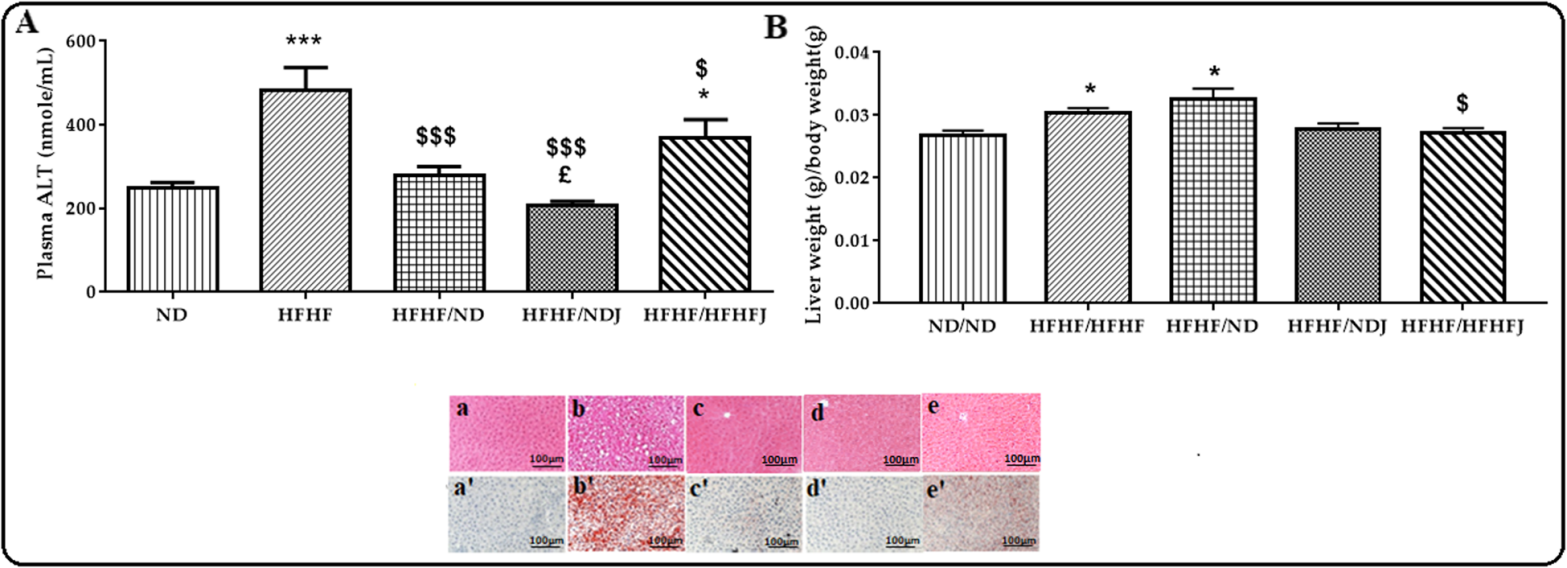
Effect of jojoba meal on liver parameters. ND (**a**, **a**’), HFHF (**b**, **b**’), HFHF/ND (**c**, **c**’), HFHF/NDJ (**d**, **d**’), HFHF/HFHFJ (**e**, **e**’). *significant difference compared to ND rats, $ significant difference compared to HFHF rats, ^£^significant difference compared between HFHF/ND rats and HFHF/NDJ rats. All values are indicative of Mean ± SD. *p < 0.05, ** *p* < 0.01, *** *p* < 0.001.

The effect of the various diets on ALT is shown in Fig. 5. After 16 weeks of HFHF diet, a significant increase (*p* < 0.001) in the ALT level was recorded (248.38 nmol/mL ±10.33 (ND) vs. 42.51 nmol/mL ±38.69 (HFHF)). The switch of diet to NDJ or HFHFJ significantly reduced ALT levels (206.36 nmol/mL ±7.50, *p* < 0.001; 386.00 nmol/mL ± 32.02 nmol/mL, *p* > 0.05). The effect was amplified with ND (280.33 ± 14.7, *p* < 0.001), (Fig. 5a).

The HFHF diet induced a significant increase in liver weight (14.6 ± 0.53 g) compared to control ND rats (12.52 ± 0.33 g), (Fig. 5b). After 2 months, the change to a jojoba rich diet either in ND or HFHF diet allowed the significant decrease (*p* < 0.01) of liver weight (HFHF/NDJ: 10.97 ± 0.41 g and HFHF/HFHFJ: 10.97 ± 0.34 g). The switch to ND did not affect liver weight.

These results were confirmed by histological study of the liver where the nuclei of the hepatocytes of the ND rats were clearly violet in a pink colored cytoplasm. This structure showed no deposit of the lipid droplets with a score 0 [21]. In contrast, a steatosis was well marked in rats fed with HFHF. In fact, the hepatocytes in these rats were flushed with vacuoles in fat droplets. The nuclei were not clear in the cytoplasm and the steatosis exceeded 85% with a score of 3 [21]. These disturbances were reversed in the other rat groups where the ND allowed a decrease of this steatosis to a score between 0 and 1.

### Effect of jojoba meal on renal dysfunction

**Fig. 6:**
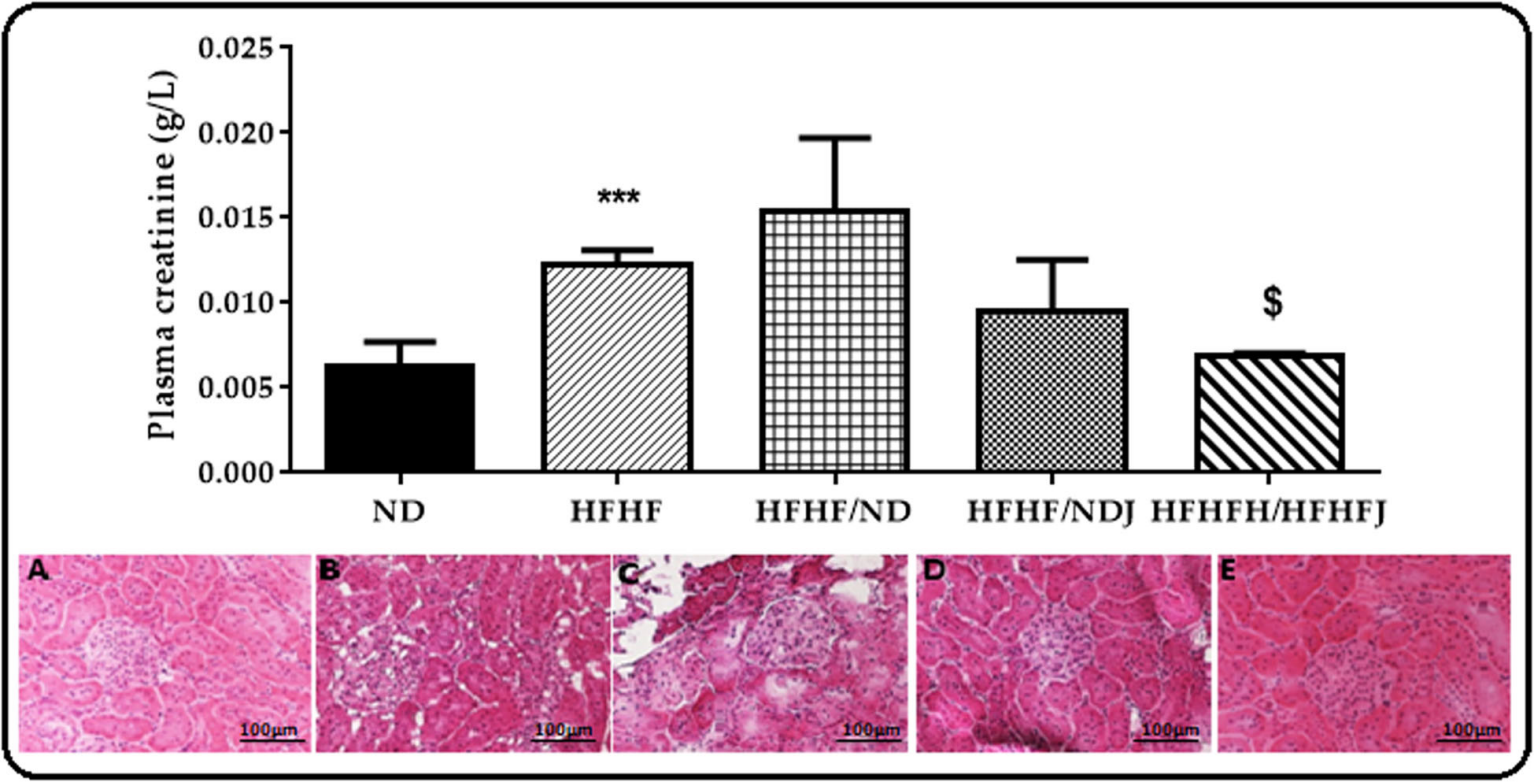
Effect of jojoba meal on plasmatic creatinine and kidney histology. *significant difference compared to ND rats, $ significant difference compared to HFHF rats. ND (**a**), HFHF (**b**), HFHF/ND (**c**), HFHF/NDJ (**d**), HFHF/HFHFJ (**e**). All values are indicative of Mean ± SD. *p < 0.05, ** *p* < 0.01, *** *p* < 0.001.

HFHF diet induced a significant (*p* < 0.001) increase in creatinine compared to ND (0.006 ± 0.00 vs. 0.12 ± 0.00 g/L). Only the treatment with HFHFJ significantly decreased the creatinine levels (0.07 ± 0.00 g/L, *p* < 0.05) (Fig. 6).

The microscopic observations of the histological kidney sections of ND rats showed a normal structure whereas it was disrupted in the HFHF rats. Indeed, an enlargement of the lighthouse and the Bowman’s space and dilation in the glomerular capillaries were observed with a fragmentation of the glomerulus, testifying of a necrosis. This disturbance was partially reversed with the ND switch where the Bowman’s space remained still a little bit larger when compared to the control group. On the other hand, the switch to NDJ or HFHFJ seemed to restore to normal state the kidney structure (Fig. 6).

### Effect of jojoba meal on oxidative stress parameters

**Fig. 7:**
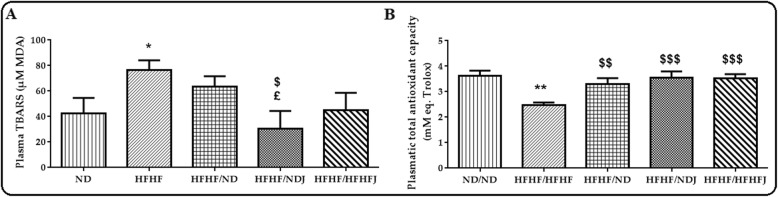
Effect of jojoba meal on plasmatic TBARS and total antioxidant capacity. *significant difference compared to ND rats, $ significant difference compared to HFHF rats, ^£^significant difference compared between HFHF/ND rats and HFHF/NDJ rats. All values are indicative of Mean ± SD. *p < 0.05, ** *p* < 0.01, *** *p* < 0.001.

HFHF diet induced a significant (*p* < 0.05) increase in TBARS levels (Fig. 7a) after 16 weeks compared to ND (76.68 ± 5.48 vs. 42.26 ± 8.92 μMMDA). Only the switch to NDJ reduced significantly (*p* < 0.05) the increase in TBARS. ND and HFHFJ appeared to not affect the TBARS levels. On the other hand, HFHF induced a significant (*p* < 0.001) decrease in TAOC (2.47 ± 0.73 (HFHF) vs. 3.62 ± 0.16 (ND) mM eq. Trolox). The switch to ND, NDJ or HFHFJ diet protected against this decrease (ND: 3.30 ± 0.20, *p* < 0.01; NDJ: 3.55 ± 0.18, *p* < 0.001 and HFHFJ: 3.51 ± 0.15 mM eq. Trolox, *p* < 0.001) (Fig. 7b).

## Discussion

The combination of fat and sugar in the diet (HFHF diet) has led to the development of a metabolic disorder leading to T2D, attested by pre and postprandial hyperinsulinemia, glucose resistance, and maintenance of fasting hyperglycemia. Addition of jojoba meal provoked an anorexic effect and demonstrated its ability to limit hyperglycemia, IR, hyperleptinemia, hepatic steatosis and associated oxidative stress.

Firstly, we developed in our study a very interesting animal model with HFHF diet. Leptinemia associated with fat storage was observed in the animals and has led to a reduction of insulin sensitivity even without weight gain. This metabolic disorder was explained by some authors as an expansion of the adipose cells [22]. In fact, IR is attested by hyperinsulinism and is the cause of deregulation of glycemic (post-prandial glycaemia) [23]. The hyperglycemia and hyperinsulinemia demonstrated in our study were then associated to oxidative stress. Leading to increased free radicals generation via multiple mechanisms. Patients with diabetes may be especially prone to acute and chronic oxidative stress which enhances the development of late diabetic complications. Oxidative stress at the cellular level subsequently induces hepatic steatosis, inflammation and fibrosis. Oxidative stress is considered a key contributor to progression from simple fatty liver to NASH. Several models have been put in place and have been associated with liver changes including hepatic steatosis. These models were presented for a diet rich in fructose [24, 25] or rich in fat [26, 27] or rich in fat and carbohydrates [28].

In our case, ALT level was measured and showed an increase in HFHF compared to ND rat groups. These results are in accordance with Castro et al. [28] who showed that the fructose and fat diets induced an increase in ALT level in adult and young rats, respectively.

According to the literature, a metabolic disorder caused by HFHF can provoke renal alterations such as accumulation of fat cells in the kidney [29]. This has been also confirmed in our study where an accumulation of fat cells in the kidneys was observed under HFHF diet. The mechanism leading to this disorder by HFHF is still poorly understood but can be explained according to Fukuzawa et al., [30] by renal glucose transporters such as Glucose transporter isoforme 5 (GLUT5), GLUT2, Sodium-Glucose Cotransporter 4 (SGLT4), Na + −dependent Glucose Transporter (NaGLT1) [31, 32] and SGLT5 which are expressed in the kidney and allow the transport of mannose and fructose [33].

In a previous study, we have already demonstrated in vitro the protective effect of Jojoba seed extracts against hyperglycemia-induced oxidative stress through the modulation of RINm5f beta cell cytotoxicity, generation of ROS, insulin release, caspase-3, activation, pro-oxidant and antioxidant defense, and status of the cells. Using this elegant in vivo insulin resistance model, our purpose is to demonstrate the efficiency of jojoba seeds [18].

First of all, addition of jojoba seeds in the animal’s diets experimented caused a loss of appetite in rats. This was also demonstrated and proven in the literature [34] reporting the anorectic effect of jojoba and its effect on weight loss. In fact, the anorectic effect was due to the presence of simmondsin molecule once orally administered in jojoba containing meal [35]. According to these authors, s the administration of Cholecystokinin A (CCKA) receptor antagonists suppressed the effect on satiety. These receptors inhibited the anorect ic effect of exogenous cholecystokinin in rats and mice [36, 37]. The increase in satiety caused by simmondsin and that of CCK is generally due to the vague nerve. Indeed, vagotomy in rats reduced the effects of exogenously administered simmondsin and CCK [34]. Marnix et al. [38] have worked with the same concentration of jojoba used in our study and the results showed that this concentration caused a reduction in the food intake and weight loss in the rats in 2 weeks. Boozer et Herron [39] proved that simmondsin produced a clear dose-response effect on this dietary intake as well as weight loss and that simmondsin at 0.15 and 0.25% significantly reduced these parameters without any undesirable effects. On the other hand, if this concentration is higher than 0.5%, many complications can be placed on the composition of white blood cells. The quantity of simmondsin in Jojoba seeds varied from 2.6–4.2 g per 100 g seeds. In our study, we add 3% of total jojoba seed in the diet (0,078 g-0,126 g for 100 g of diet). This level is very low but sufficient to reduce body weight without apparent negative effects.

Treatment by ND or jojoba meal allowed regulating the glucose tolerance. There is no work done on jojoba meal in this context and consequently the explanation of this phenomenon remains always little known. Indeed, the response of insulin to the pancreas and consequently to its sensitization to glucose has been admitted, and it has been shown that the pancreas is inactivated by the autonomic nervous system and that the hypothalamus regulates this sensibility by the means of this innervation of the autonomic nervous system [40]. La Fleur et al. [41] showed that in the arcuate nucleus, proopiomelanocortin mRNA expression and neuropeptide Y were changed in High Fat High-Fructose diet encouraging IR and glucose intolerance. These two neurons in the arcuate nucleus touch the glycated metabolism by the hypothalamic neurons which control the autonomic nervous signal of several organs including the pancreas [40]. Therefore, changes and alterations in the brain caused by HFHF diet can affect the metabolism of glucose.

Another explanation for the reduced IR after eating jojoba meal was played on the energy balance by decreasing food intake. In fact, in response to the lack of calories, or on the contrary to supercharging, the activation of signaling channels for stimulation and feedback ensures that changes in the energy balance are triggered. In skeletal muscle in rodents, calories restriction particularly increases the transport of glucose [42].

The treatment with jojoba meal made it possible to remove these lipid droplets which show the non-toxicity of the jojoba meal on the kidney. Our results were in agreement with Boozer and Herron [39] who did not find a difference between the tissue structures of the kidneys of the untreated and simmondsin treated rats.

The increase of ALT level by HFHF diet was reversed by ND and jojoba meal while staying on a diet without high-fat high-fructose or on diet mixed with HFHF diet. Our results were in agreement with Abdel-Wahhab et al. [43]. These authors showed a decrease in the ALT level by ethanolic extract. They also testifyied that this treatment was not toxic to the liver in rats fed with fumonisin B1-contaminated diet.

A regulation of oxidative stress towards TBARS was achieved by the jojoba treatment associated with an increase in the TAOC. There is no former work dealing about the neo-oxidative power of jojoba on the metabolic syndrome and that would explain this antioxidant capacity improvement. Nevertheless, several authors have demonstrated the richness of jojoba meal in antioxidant molecules such as tannins [44, 45], anthocyanins such as malvidin [46] and alkaloids [47] providing a strong argument to explain the observed positive effects against oxidative stresses.

## Conclusions

This study elucidated the protective effects of jojoba seeds in a metabolic syndrome rat model. We observed this treatment had significant effects on plasma and tissues (pancreas, kidney and liver) with a proved anorexic effect.

The anorexic effect of jojoba seed is due to leptin as proven by other authors [34] by its central action on the hypothalamus by activating AMP kinase (AMPK) in muscle and liver. As a result, leptin and consequently the jojoba seed acts on multiple peripheral tissues and modules in particular the glucido-lipid metabolism by these actions on beta-pancreatic islets, adipose tissue, muscle and liver by improving significantly insulin sensitivity in both the liver and muscle with a major regression of fatty liver disease.

The latter presents one of the interesting parts of our study which is due to weight lost produced by the anorexic effect of jojoba but also to specific antioxidative properties (TBARS and total capacity antioxidant) of the jojoba seeds, confirming in vivo the previous data obtained in vitro on RIN-m5f beta cell lines [18].

However, one of the limitations could be due to the duration of the study. It would be interesting to test this concentration on an animal model where the type 2 diabetes onset would last for longer period. Moreover, the other limitations could be attributed to the total Jojoba seeds used in the study. It is not possible to determine which molecule is causing one effect over another. Even if the total beneficial effect observed in this study can be attributed to the synergistic effect of the different molecules present in the jojoba seeds, it would be interesting to study more precisely the effect of purified extract other than Simmondsin as it has already been done extensively. in the literature.

## Supplementary information

**Additional file 1.**

## Data Availability

Data sharing not applicable to this article as no datasets were generated or analyzed during the current study.

## Abbreviations

ALT: Alanine transerase
DT2: Type 2 diabetes
GLUT5: Glucose transporter isoforme 5
HDL: High Density lipoproteins-cholesterol
HFHF: High Fat and High Fructose diet
HFHFJ: High Fat and High Fructose and Jojoba diet
HOMA2-IR: Homeostasis Model Assessment Indexes-Insulin Resistance
IpGTT: Intraperitoneal glucose tolerance test
NaGLT1: Na + −dependent Glucose Transporter
NASH: Non-Alcoholic Steatohepatitis
NDJ: Normal Diet Jojoba
OS: Oxidative stress
ROS: Reactive Oxygen Species
SGLT4: Sodium-Glucose Cotransporter 4
TAOC: Total Antioxidant Capacity
TBARS: ThioBarbituric Acid Reactive Substances
