# Supplementary figures and images for: Anorexic and metabolic effect of jojoba: potential treatment against metabolic syndrome and hepatic complications
Source: Nutr Metab (Lond). 2020 Mar 30;17:24. doi: 10.1186/s12986-020-00441-3 (PMC7106724; doi:10.1186/s12986-020-00441-3)

S1


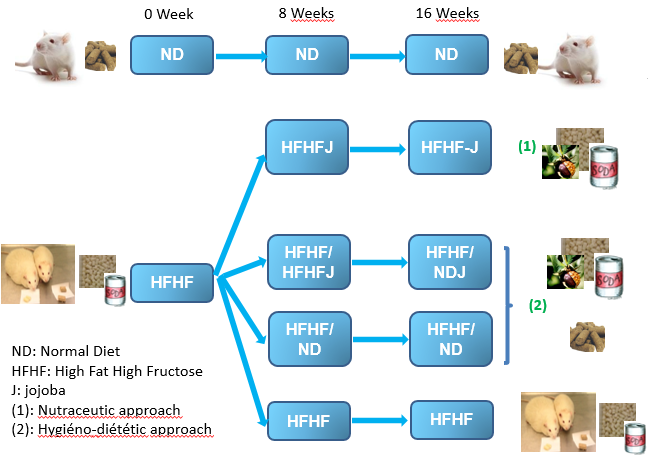

Supplement: Supplementary file 1 — Additional file 1. [file 12986_2020_441_MOESM1_ESM.docx]
